# WALKING CADENCE AS A MEASURE OF ACTIVITY INTENSITY AND THE IMPACT ON FUNCTIONAL CAPACITY FOR OLDER ADULTS WITH FRAILTY

**DOI:** 10.1093/geroni/igad104.3693

**Published:** 2023-12-21

**Authors:** Daniel Rubin, Anthony Hung, Donald Hedeker, David Conroy, Megan Huisingh-Scheetz, Jennifer Brach, Nancy Glynn, Margaret Danilovich

**Affiliations:** University of Chicago, Chicago, Illinois, United States; University of Chicago, Chicago, Illinois, United States; University of Chicago, Chicago, Illinois, United States; Penn State University, University Park, Pennsylvania, United States; University of Chicago, Chicago, Illinois, United States; University of Pittsburgh, Pittsburgh, Pennsylvania, United States; University of Pittsburgh School of Public Health, Pittsburgh, Pennsylvania, United States; Northwestern University, Chicago, Illinois, United States

## Abstract

**Background:**

Walking cadence has been suggested as a measure of activity intensity; however, it remains uncertain if prefrail and frail older adults can increase their walking cadence and if doing so leads to improvements in functional capacity. We aimed to determine if cadence can be increased and if this leads to improvement in functional capacity in prefrail and frail older adults.

**Methods:**

We performed a secondary data analysis of a walking intervention in prefrail and frail older adults living in retirement communities. Patients were randomized to Casual Speed Walking (CSW) and High-Intensity Walking (HIW) groups. Our primary outcome was improvement in 6-minute walk test (6MWT) distance above the minimally clinical important difference (MCID). We performed linear and logistic mixed-effects regressions to analyze our aims.

**Results:**

102 participants were included in the final analysis with 56 in the CSW group and 46 in the HIW group. Participants in the HIW group increased their walking cadence as compared to the CSW group during the intervention (HIW 100[88, 111] steps/min vs. CSW 77[65, 86] steps/min; P < 0.001). Participants that increased their walking cadence demonstrated an increased odds of improvement in their 6MWT MCID (OR: 0.11, 95% CI: 0.033, 0.18; p=0.005).

**Conclusions:**

Older adults can increase their walking cadence and walking cadence can serve as a surrogate measure of activity intensity during walking interventions. An increase of 14 steps/minute from their comfortable walking cadence increased the odds of improvement in 6MWT MCID.

